# Claudin-3 expression increases the malignant potential of lung adenocarcinoma cells: role of epidermal growth factor receptor activation

**DOI:** 10.18632/oncotarget.14974

**Published:** 2017-02-01

**Authors:** Lianmin Zhang, Yuan Wang, Bin Zhang, Hua Zhang, Meng Zhou, Mei Wei, Qiuping Dong, Yue Xu, Zhaosong Wang, Liuwei Gao, Yanjun Qu, Bowen Shi, Jinfang Zhu, Yuesong Yin, Yulong Chen, Lu Sun, Wei Zhang, Shilei Xu, Guoguang Ying, Changli Wang

**Affiliations:** ^1^ Tianjin Medical University Cancer Institute and Hospital, National Clinical Research Center for Cancer, Key Laboratory of Cancer Prevention and Therapy, Tianjin, 300060, China

**Keywords:** adenocarcinoma, tight junction, claudin-3, EGF, proliferation

## Abstract

Claudins are essential for the formation and maintenance of tight junctions (TJ). The altered expression of claudin proteins has been described in a variety of malignancies. However, the alteration of these proteins in lung adenocarcinoma (ADC) are poorly understood. Therefore, we report, based on the protein expression analysis of a total of 275 patient samples, that claudin-3 (*CLDN3*) expression is significantly increased in ADC tissues and is associated with cancer progression, correlating significantly with the poor survival of ADC patients (*p*=0.041&0.029). More importantly, forcing *CLDN3* expression in ADC cells without endogenous *CLDN3* expression resulted in significant increases in the cell proliferation, anchorage-dependent growth, migration and drug-resistance. In addition, epidermal growth factor (EGF) signaling pathway modulates the expression of claudins in a number of solid tumors. However, the mechanism of tight junction regulation by EGF in ADC remains unclear. To investigate this mechanisms, ADC cell lines were treated with EGF and its inhibitor. EGF unregulated *CLDN3* expression via the MEK/ERK or PI3K/Akt signaling pathways and was required for the maintenance of baseline *CLDN3* expression. Furthermore, downregulation of *CLDN3* expression in ADC cell was found to prevent the EGF-induced increase in cell proliferation. In conclusion, our results demonstrate a novel role of C*LDN3* overexpression in promoting the malignant potential of lung adenocarcinoma. This function is potentially regulated by the EGF-activated MEK/ERK and PI3K-Akt pathways.

## INTRODUCTION

Lung cancer remains the major cause of cancer-related deaths worldwide [1]. Lung adenocarcinoma is the most common histological subtype which is increasing in prevalence [2]. Appearance of EGFR tyrosine kinase receptor inhibitors (TKI)had brought a revolutionary change in non-small cell lung cancer(NSCLC), such as gefitinib, erlotinib, afatinib and AZD9291, especially those with adenocarcinomas [3]. EGF-driven cell signaling contributes to unregulated progression and cancer malignancy.

Claudins are a family of the major integral membrane proteins forming the backbone of tight junctions, which has been identified by the date of varying cell and tissue-specific expression level [4, 5]. The alteration of claudin expression has been reported in tumors isolated from colon [6], breast [7, 8], ovary [9], pancreas [10], prostate [11], stomach [12] and lung tissues [13]. The certain member of this family affects the development as well as the invasion and aggressive growth of cancer cells. In lung adenocarcinoma patients, the downregulation of *CLDN1* was predicted a poor survival [14]. Furthermore, the excessive expression of *CLDN1* attenuated the invasive and migrate properties of cultured ADC cells lacking endogenous *CLDN1* expression. In lung cancer tissue microarrays, *CLDN7* expression has been found to be either down-regulated or disrupted in its distribution pattern in cancerous tissue compared to normal tissue [15]. The overexpression of *CLDN7* inhibits cell migration and invasion in NCI-H1299 lacking endogenous *CLDN7* expression. *CLDN*2 expression in lung adenocarcinoma tissue is higher than that in normal tissue and other lung carcinomas. Ikari recently reported that *CLDN*2 enhances cell colonization and migration in lung adenocarcinoma cell line A549, and *CLDN*2 may be involved in the regulation of cancer cell motility [16, 17]. More importantly, *CLDN3* is involved in the development of acinus and the differentiation of alveolar epithelial cells [18] and its levels varied in different subtypes of lung carcinomas [13, 19]. Furthermore, compared to lung squamous cell carcinoma (SqCC), an increased expression of *CLDN3* was found in ADC [19–21]. There might be an association between the overexpression of *CLDN3* and the carcinogenesis of ADC. *CLDN3* and *CLDN*4 are highly overexpression in all subtypes of epithelial ovarian cancers, and these regulation enhances angiogenic effects and invasive properties as well as increased matrix metalloproteinase-2 activity [6]. In addition, *CLDN3* is one of the natural receptor for the a cytolytic toxin, *Clostridium perfringens* enterotoxin (CPE), binding to its receptor to induce cell apoptosis [22]. However, the underlying mechanisms that regulate the function and expression of *CLDN3*, particularly in lung adenocarcinoma, are poorly understood.

The expression and localization of claudin protein affect cell function, and EGF signaling pathway plays a pivotal role in the regulation of claudin protein. For instance, EGF was mediated the upregulation of *CLDN*1, 3 and 4, and downregulation of *CLDN*2, which increased the force of intercellular barrier in MDCK-II cells [23, 24]. In addition, EGF may also regulate the expression functions of claudins in ovarian and colon cancer cells during cancer development via the EGF-activated ERK1/2 and PI3K-Akt pathways [25, 26]. However, its regulatory mechanism in ADC remains unclear. Therefore, it is important to understand the expression and function of *CLDN*3 as well as molecular mechanisms regulating the expression of *CLDN*3 in ADC.

## RESULTS

### *CLDN3* expression is significantly upregulated in lung adenocarcinoma and an independent predictor for survival in ADC patients

**Figure 1:**
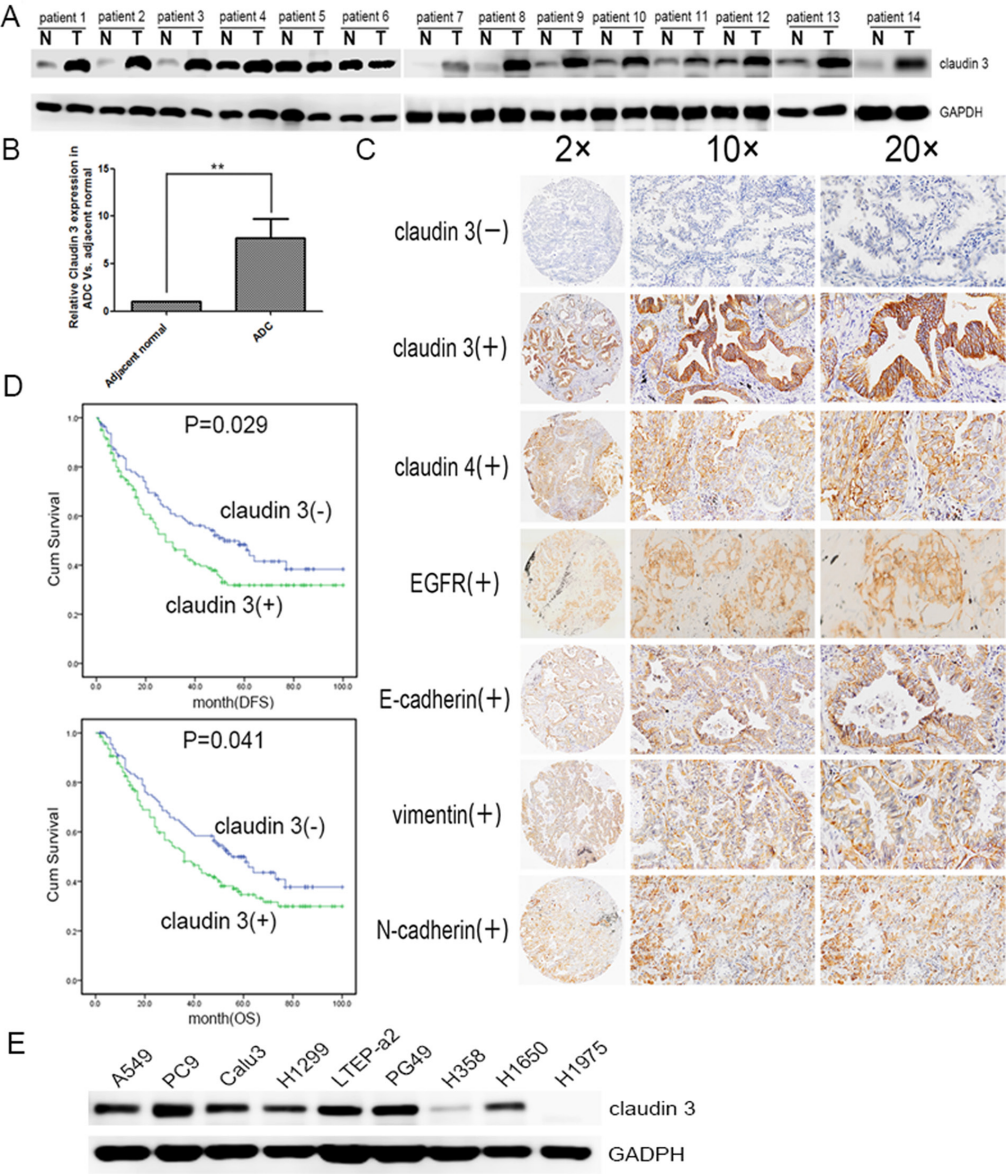
*CLDN3* expression in lung cancer patients and cell lines **A**. *CLDN3* expression in lung cancer and adjacent normal lung samples. Total tissue lysates were prepared using frozen matched normal and cancer lung tissues from the same patient. GAPDH was used as a loading control. (N, adjacent normal tissue;T, tumor tissue) **B**. *CLDN3* was significantly increased in the adenocarcinoma group compared with the normal specimens (**p<0.01). **C**. Normalized expression values for *CLDN3* in normal adjacent lung specimens (n=40) compared with adenocarcinoma samples (n=261) from the Tianjin Medical University Cancer Hospital and Institute using microarray analysis. The demographics for this group are listed in Table 1. Representative images of protein expression in an ADC determined by immunohistochemistry (IHC) with anti-*CLDN3*, anti-*CLDN4*, anti-EGFR and EMT index antibody (*brown*). The slide was counterstained with hematoxylin. Original magnification, ×20(*left*); ×100 (*middle*); ×200 (*right*). **D**. Kaplan–Meier curves for the disease free survival and overall survival rates of patients with ADC according to the expression level of *CLDN3*. *Blue*, patients with lower *CLDN3* expression (n=117); *green*, patients with higher expression of *CLDN3* (n=144, median DFS was 52-month vs. 28-month and median OS 55-month vs. 36-month, respectively; *p_DFS_*=0.029, *p_OS_* =0.041). **E**. *CLDN3* expression was detected in lung adenocarcinoma cell lines: LTEP-a 2, PC9, and PG49 cells express *CLDN3* very highly; NCI-H1650, NCI-H1299, A549, and CaLu-3 cells express moderate levels of *CLDN3*; and H1975 and H358 cells almost do not express *CLDN3* at all.

To determine the level of *CLDN3* protein in ADC tissues, we detected the expression levels between paired adjacent normal tissues and ADC specimens (n=14). We found that the level of *CLDN3* protein was significantly higher in the adenocarcinoma specimens compared with normal tissues (Figure 1A & 1B, **p<0.01). We next performed immunohistochemical analysis in 261 ADC patients using an anti-*CLDN3* antibody. The intensity of *CLDN3* staining was independently scored by two pathologists, and low and high classification was distinguished (see methods). *CLDN3* expression was lower in most of the normal lung tissue, but highter in the ADC tissues (overexpression rate 10% vs. 55%, *p*<0.01). Furthermore, The *CLDN3* protein level was significantly correlated with the *CLDN4*, E-cadherin, N-cadherin, and vimentin expression levels (*p*=0.006, *p*=0.023, *p*=0.021, and *p*=0.017, respectively), except EGFR.

The correlation between the *CLDN3* expression status and the clinicopathologic features of 261 ADCs was further evaluated, and the findings are summarized in Table 1. A positive correlation was observed between the *CLDN3* upregulation and recurrence and/or metastasis (Table 1). Furthermore, Kaplan–Meier survival analysis demonstrated that the 5-year survival rate was significantly lower in patients with *CLDN3* upregulation (n=144) than in the patients with lower *CLDN3* expression (n=117, median DFS was 28-month vs. 52-month and median OS was 36-month vs. 55-month) (*p*_DFS_=0.029; *p*_OS_=0.041, Figure 1D). Moreover, multivariate analysis showed that the upregulation of *CLDN3* (*p*_DFS_=0.020, *p*_OS_=0.018) was an independent prognostic predictors for ADC patients (Table 2).

**Table 1:** Association between *CLDN3* expression and clinicopathologic characteristics of patients with ADC

Characteristics	No	Claudin3 (+)	Claudin3(−)	χ^2^*P*
Age (Years)				0.319
≤60	143	74(51.7%)	69(48.3%)	
>60	118	69(58.5%)	49(41.5%)	
Gender				0.305
Male	124	70(56.5%)	54(43.5%)	
Female	137	73(53.3%)	64(46.7%)	
Smoking history				0.910
Never	140	76(54.3%)	64(45.7%)	
Ever	121	67(55.4%)	54(44.6%)	
Tumor size (cm)				0.275
≤ 3	134	68(50.7%)	66(49.3%)	
>3	127	74(61.2%)	53(38.8%)	
T stage				0.368
T1	118	59(50%)	59(50%)	
T2	88	53(60.2%)	35(39.8%)	
T3	55	31(56.4%)	24(43.6%)	
N stage				0.144
N0	140	74(52.9%)	66(47.1%)	
N1	31	23(74.2%)	8(25.8%)	
N2	90	46(51.1%)	44(48.9%)	
TNM stage				0.551
I(IA, IB)	121	65(53.7%)	56(46.3%)	
II(IIA, IIB)	52	32(61.5%)	20(38.5%)	
IIIA	88	46(52.3%)	42(47.7%)	
Histology				
Micropapillary predominent	33	19(57.6%)	14(42.4%)	0.423
Acinar predominent	29	19(65.5%)	10(34.5%)	0.513
Lepidic predominent	99	49(49.5%)	50(50.5%)	0.029
Solid predominent	78	44(56.4%)	34(43.6%)	0.970
Papillary predominent	22	16(72.7%)	6(26.3%)	0.509
Recurrence or Metastasis				0.008
Absent	146	69(47.3%)	77(52.7%)	
Present	115	74(64.3%)	41(35.7%)	

**Table 2:** Association of various factors with DFS and OS in 261 ADCs determined by COX regression model

Variable		Multivariate analysis for DFS		Multivariate analysis for OS	
		HR_a_(95%CI_b_)	*P*_c_	HR(95%CI)	*P*
Gender	Male vs. Female	0.887(0.612-0.228)	0.530	0.913(0.627-1.331)	0.637
Age	>60yr vs. ≤60yr	0.947(.680-1.319)	0.748	0.973(0.698-1.356)	0.872
Smoking history	Never vs. Ever	1.589(1.093-2.311)	0.015	1.594(1.092-2.326)	0.016
TNM stage	Early vs. Advanced	1.657(1.365-2.011)	0.001	1.712(1.406-2.084)	0.001
Chemotherapy	yes vs.no	1.076(0.739-1.567)	0.702	0.945(0.647-1.381)	0.772
Radiotherapy	yes vs.no	1.463(0.952-2.249)	0.083	1.365(0.886-2.102)	0.158
*CLDN3* expression	Low vs. High	1.491(1.065-2.087)	0.020	1.501(1.072-2.101)	0.018

_a_HR: hazard ratio for death.

_b_CI: Confience interval.

_c_*P* <0.05 was considered statistically signifiant.

Similarly, *CLDN3* was also significantly upregulated in 7/9 (78%) of the ADC cell lines (A549, PC9, CaLu-3, NCI-H1299, LTEP-a 2, PG49, NCI-H358, NCI-H1650 and NCI-H1975, Figure 1E). Taken together, our data demonstrates significant *CLDN3* overexpression in ADC tissues and predicts a potential correlation between *CLDN3* expression and cancer progression.

### Alterations in *CLDN3* expression affect the proliferation, clonality, migration and protect against the effects of cisplatin

**Figure 2:**
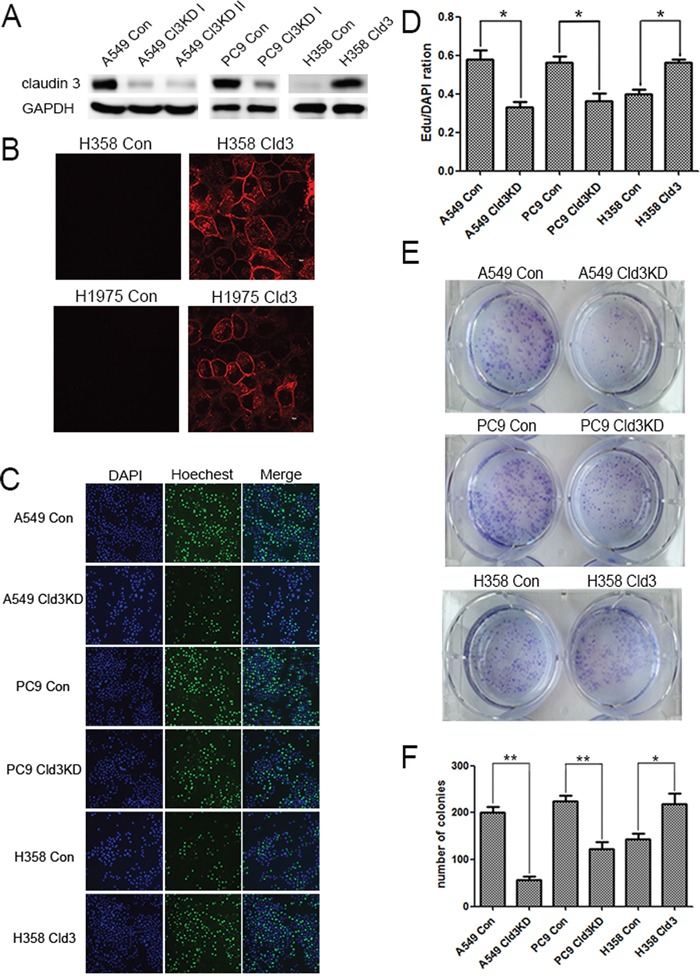
*CLDN3* expression alterations affect the proliferation and clonality of ADC cells **A**. A549 and PC9 cells were infected with a control lentivirus or lentivirus-expressing shRNA specific to *CLDN3* and then selected with puromycin. NCI-H358 cells transfected with either the control vector or *CLDN3* was analyzed by western blotting. **B**. Confocal image of the immunofluorescence staining using an anti-*CLDN3* antibody. The expressed *CLDN3* is localized at the apical cell membrane along with minor cytoplasmic distribution. Scale bar, 10 μm. **C&D**. Cell proliferation in A549(Cld3KD), PC9(Cld3KD), NCI-H358^Cld3^cells and the respective control cells using the EDU assay (*p<0.05, t-test). (D) Representative photographs of anchorage-dependent colonies that were stained with crystal violet. **E**. The bar graphs show the number of colonies increase in foci formation of ectopic expression of *CLDN3* to the control cells (*p<0.05, ** *p* <0.01, t-test).

**Figure 3:**
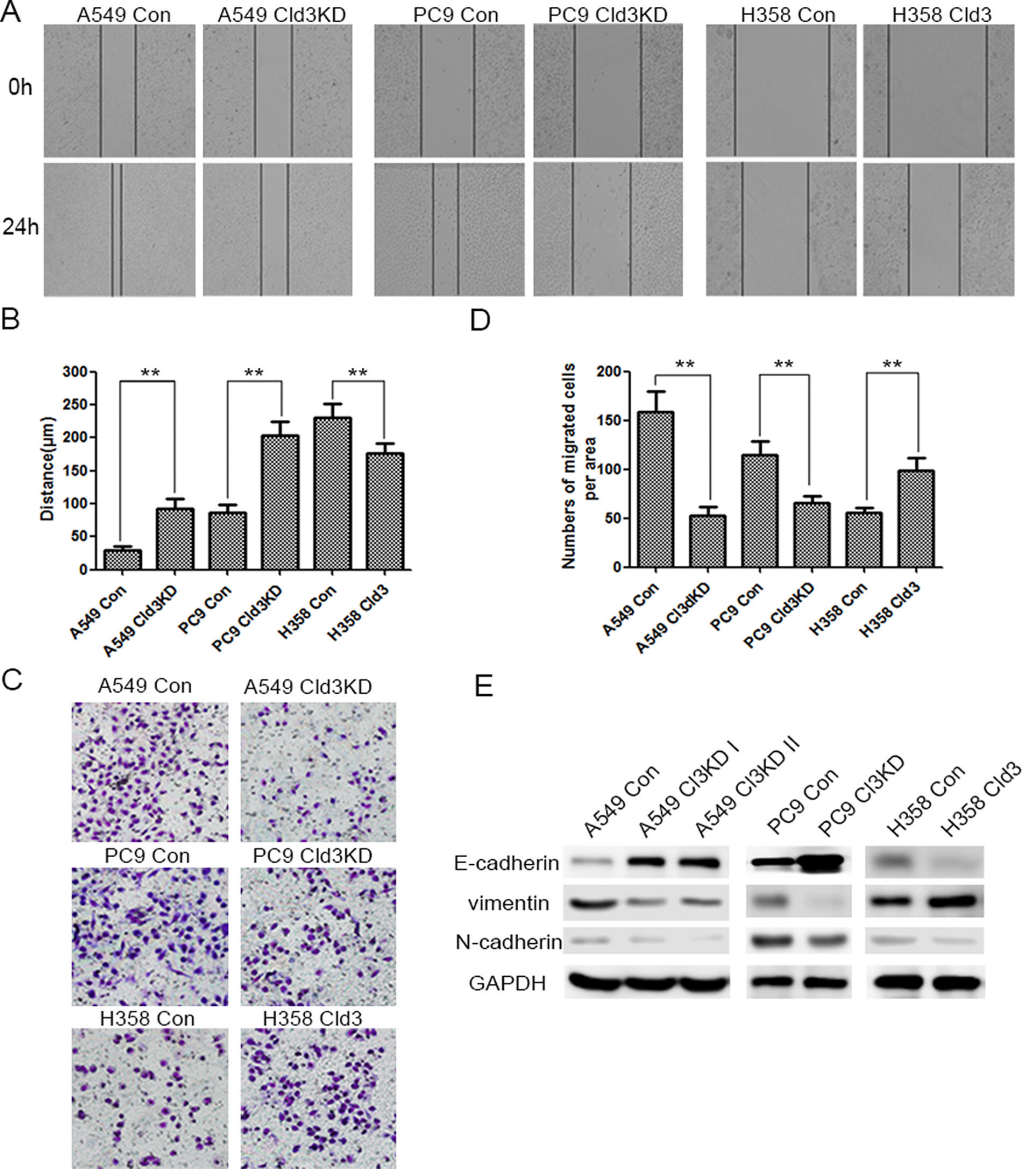
*CLDN3* expression alterations affect the migration of ADC cells **A&B**. The effect of *CLDN3* on cell migration was determined by a wound-healing assay. During a period of 24 h, the spreading speed of Cld3KD expressing cells (A549 Cld3KD/PC9 Cld3KD) along the wound edge was slower than that in control cells (A549 Con/PC9 Con), while the overexpression of *CLDN3* in the H358 cells promoted cell mobility. (** *p* <0.01, t-test) **C**. Similarly, the ectopic expression of *CLDN3* significantly enhanced the migration ability in H358 cells, while the knockdown of *CLDN3* by shRNA significantly inhibited the migration both in the A549 and PC9 cells. **D**. The number of migrated tumor cells is quantified in the below panel. (**p<0.01, t-test). **E**. The expression of epithelial or mesenchymal markers in NCI-H358 cells transfected with either the control vector or *CLDN3* was analyzed by western blotting. GAPDH was used as a loading control. The upregulation of *CLDN3* initiated EMT *in vitro*.

As shown in Figure 2, in the *CLDN3* downregulation condition (Figure 2A), we observed decreases in proliferation both in the A549 (Cld3KD) and PC9 (Cld3KD) cells (Figure 2C, & 2D; *p<0.05, **p<0.01). Conversely, in the *CLDN3* overexpression condition(Figure 2A & 2B), we observed increased proliferation in H358**^Cld3^** compared to their respective controls (Figure 2C & 2D; *p<0.05). It is well known that cell transformation plays an important role in evaluation of malignant potential [27]. In this context, we evaluated the cell-transforming potential of *CLDN3* in A549, PC9, and NCI-H358 cells. As observed in Figure 2E, the *CLDN3* upregulation group displayed more numbers of anchorage-dependent colonies compared with control group, while the downregulation of *CLDN3* led to the reverse trend (Figure 2D & 2E, **p<0.01; *p<0.01). Furthermore, the wound healing assay and transwell system were used to examine the migration ability of the cells. Twenty-four hours after seeding, the monitored wound closure and transwell system showed that the overexpression of *CLDN3* promoted migration, while downregulation of *CLDN3* significantly restrained cell migration (Figure 3A, 3B, 3C & 3D, **p<0.01). As shown in Figure 3E, the level of *CLDN3* was correlated with E-cadherin, N-cadherin, and vimentin expression. The results above suggest that CLDN3 acted as an oncogene in human lung adenocarcinoma, and *CLDN3* overexpression significantly increased the tumorigenicity and development of both cell types under study.

**Figure 4:**
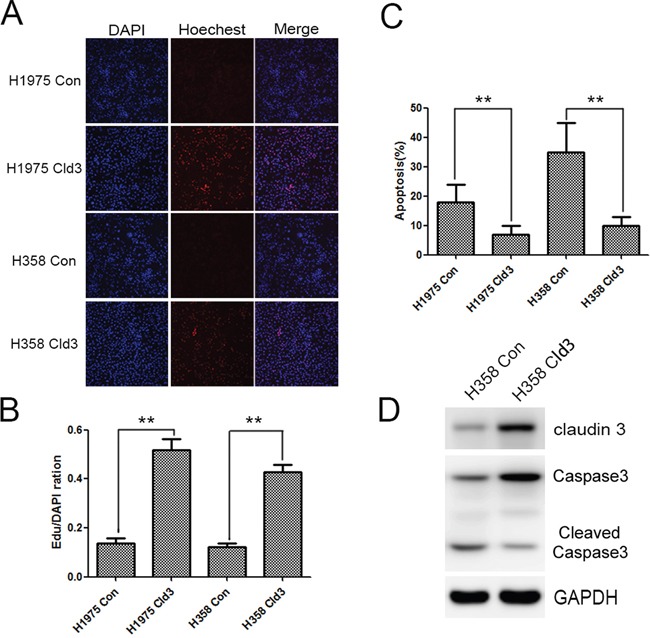
*CLDN3* overexpression protects against the effects of cisplatin **A&B**. Effects of cisplatin on the cell viability of the H1975**^Cld3^**, H358**^Cld3^** and respective control cells. Cell viability was determined at 24 hours after exposure to cisplatin. (**p <0.01, t-test). **C**. AnexinV-PI apoptosis analysis. Data were expressed as mean±SEM from three independent experiments (**p <0.01, ANOVA). **D**. *CLDN3* overexpression protects against cisplatin cytotoxicity, as indicated by decreased caspase3 degradation.

Furthermore, we detected the effects of cisplatin, a traditional lung cancer treatment drug, on the H1975**^Cld3^**, H358**^Cld3^** and control cells. Cells were exposed to a given concentration of cisplatin (4μg/ml), and cell viability was examined by EDU assay after 24 hours. As expected, cisplatin decreased the cell viability, and the H1975**^Cld3^** and H358**^Cld3^** cells were significantly insensitive from the cisplatin compared to the control groups (Figure 4A & 4B, **p<0.01). In addition, we determined that cisplatin treatment inhibited proliferation while increasing apoptosis (Figure 4C, **p<0.01) in both of the control group cells, and increased caspase3 degradation in control group cells compared with H358**^Cld3^** cells.

### EGF mediated increasing the protein levels of *CLDN3* in NCI-H358 and NCI-H1975 Cells

**Figure 5:**
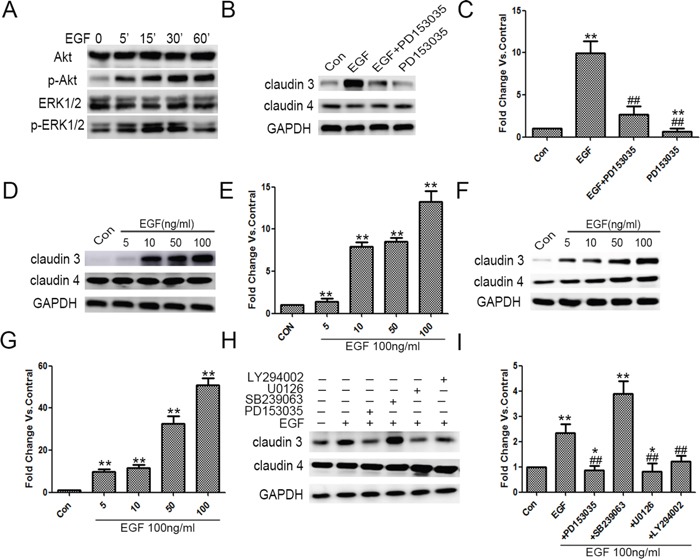
Exogenous EGF increased *CLDN3* expression in ADC cells, and this effect was mediated through ERK 1/2 and PI-3 Kinase **A**. A549 cells were grown and treated with EGF for 5, 15, 30 and 60 min, after which the total cell lysates were harvested and analyzed by immunoblotting for p-ERK1/2, ERK1/2, p-Akt, and Akt. **B&C**. Quiescent A549 cells were exposed to EGF (100 ng/ml), PD153035 (0.5 μM) or both. The effect upon *CLDN3*, *CLDN4* expression was examined. GAPDH was used for normalization (**p<0.01 EGF treated vs. control, ^##^ *p*<0.01 EGF treated vs. EGF +PD153035, and **p<0.01 PD153035 alone vs. control, ANOVA). **D-G**. EGF-induced increase in *CLDN3* expression was dose-dependent in NCI-H1975 and NCI-H358. (**p<0.01 EGF treated vs. control, ANOVA). **H**. Quiescent A549 cells were exposed to EGF alone or in combination with PD153035 (0.5 μM), U0126 (10 μM), SB239063 (5 μM) or LY294002 (20 μm). The effect upon *CLDN3* and *CLDN4* expression was determined. GAPDH was used as a loading control. **I**. Representative densitometric analysis (*p<0.05 vs. control, ** *p* <0.01 vs. control and ^##^ *p*<0.01 vs. EGF treated, ANOVA).

Initially, we examined that EGF treatment significantly induced the alteration of the protein levels of EGFR downstream p-Akt and p-ERK1/2 in a time-dependent manner (Figure 5A). In addition, EGF treatment also mediated an increast expression of *CLDN3*, which was inhibited by EGF inhibitor, PD153035. More importantly, PD153035 treatment alone inhibited the basal level of *CLDN3* in ADC cells that did not receive EGF treatment, suggesting that EGFR signaling pathway activation is potential required for the maintenance of *CLDN3* expression (Figure 5B &5C, compared with control cells, **p<0.01; compared with EGF-treated cells, ^##^*p*<0.01). Furthermore, EGF-activated increase in *CLDN3* expression was dose-dependent both in the NCI-H358 and NCI-H1975 cells, while the levels of *CLDN4* was not changed in this same manner (Figure 5D–5G, **p<0.01). These results demonstrated that EGF activation differentially regulates the claudin members depending on the cellular context.

### EGF-activated MEK/ERK and PI-3K/AKT signaling pathways mediate the increase in *CLDN3* expression

EGFR-activated downstream signaling pathways, ERK1/2 kinase, p38 MAP kinase and/or PI-3 kinase, play key roles in the regulation of EGFR-dependent cellular functions. We further made use of pathway-specific inhibitors, for which cells were pretreated with either SB239063 (p38 MAP kinase inhibitor), U0126 (MRK kinase inhibitor), or LY294002 (PI -3 kinase inhibitor). The SB239063 had no effect on either EGF-induced or the baseline *CLDN3* expression, while the LY294002 partially inhibited the EGFR- induced increase in the *CLDN3* expression (^##^*p*<0.01, EGF+LY294002 vs. EGF-treated). Notably, the U0126 inhibited the EGF-dependent upregulation and basic line expression of *CLDN3*, which is similar to EGFR inhibitor (Figure 5H; **p<0.01, compared with control, ^##^*p*<0.01, EGF+PD153035 or EGF+U0126 vs. EGF treated, and *p<0.05 EGF+PD153035 or EGF +U0126 vs. control). Taken together, our data indicated the critical role of the EGFR and its downstream ERK1/2 kinase in the regulation of *CLDN3* expression.

### Genetic silencing of *CLDN3* expression prevents EGF-induced proliferation in ADC cells

**Figure 6:**
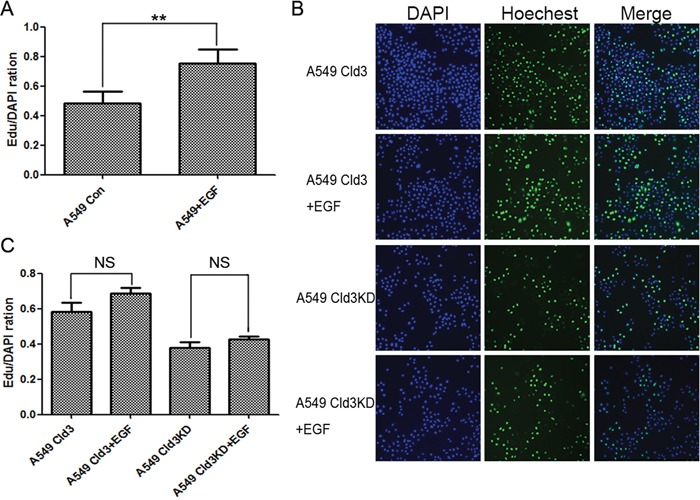
*CLDN3* expression depends on the EGFR tyrosine kinase activity and modulates EGF-induced cell proliferation **A**. Effects of EGF treatment on cell proliferation (**p<0.01, t-test). **B&C**. Effects of EGF-treatment on cell proliferation in A549^Cld3^ and A549^Cld3KD^ cells. All values presented are the mean + SEM (NS, not significant, t-test).

We further demonstrated EGF treatment (100 ng/ml) significantly increased the cell proliferation in ADC cells (Figure 6A, **p<0.01). In addition, we either silenced *CLDN3* expression (A549Cld3KD cells) or stably overexpressed it (A549^Cld3^ cells) to examine the relationship between EGF-mediated *CLDN3* expression and cell proliferation. Further studies showed significant increase in the proliferation of A549^Cld3^ cells (*p*<0.05, data not shown) and a decrease in A549 (Cld3KD) cells (*p*<0.05, Figure 4A) compared to the control groups, which supported our findings from H358 cells (Figure 4). More importantly, further studies, in which either A549^Cld3^ or A549 (Cld3KD) cells were exposed to EGF treatment (100 ng/ml), illustrated that upregulation of *CLDN3* did weaken the EGF-induced cell proliferation, and the genetic silencing of *CLDN3* expression prevented the EGF-induced proliferation in ABC cells (Figure 6B & 6C)

### Downregulation of *CLDN3* inhibits tumor growth in nude mice

**Figure 7:**
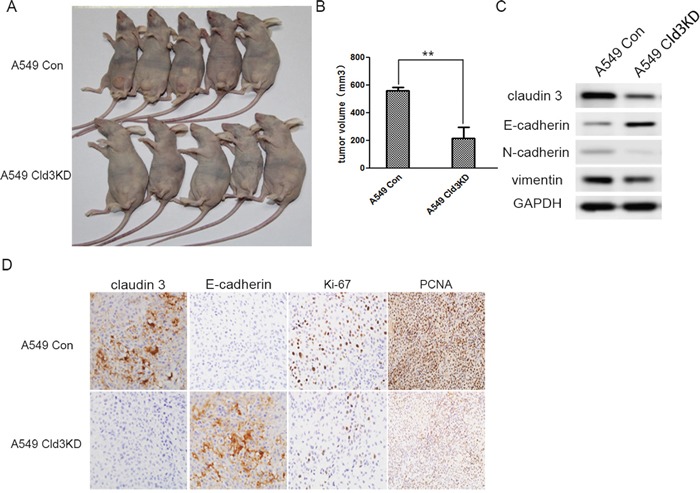
*In vivo* tumor promoting role of *CLDN3* in ADC **A**. Representative examples of tumors formed in nude mice following the injection of *Cld3KD* expressing A549 cells (*lower panel*) and A549 Con cells (*upper panel*). **B**. The volume of A549^Cld3KD^ group is much smaller than control group(**p<0.01, t-test) **C**. The knockdown of *CLDN3* in A549 cells induced E- cadherin upregulation, but decreased vimentin and N-cadherin expression **D**. Compared with the control group, the *Cld3KD* group demonstrated upregulation of E-cadherin and a lower proliferation rate (Ki-67 and PCNA index).

To further examine the tumor proliferation ability of *CLDN3 in vivo*, tumor formation in the nude mouse model was tested by injecting A549 (Cld3KD) cells (n=7) using A549 Con cells (n=7) as controls. Within 25 days, solid tumors were readily visible in the left hind legs of the 10 mice injected with the A549 (Cld3KD) cells and the control groups, respectively (Figure 7A). Moreover, the size of the tumors caused by the A549 (Cld3KD) cells was significantly smaller than those tumors induced by the A549 Con cells (** *p*<0.01, Figure 7B). Furthermore, the downregulation of *CLDN3* also had an impact on the expression of E-cadherin and vimentin *in vivo* (Figure 7C), and A549 (Cld3KD) cells showed a lower proliferation rate compared with the control groups (Figure 7D, *p* <0.01), consistent with the *in vitro* findings. These results demonstrated that *CLDN3* closely associated with tumor proliferative capacity *in vivo*, and probably played a pivotal role in EMT process.

## DISCUSSION

*CLDN3* belongs to a family of proteins important in TJ formation and function. Like the tumors in other organs, lung tumors also demonstrate variation in the expression in TJ proteins [28]. For instance, *CLDN5* expression was detected in most of adenocarcinomas, but squamous cell carcinomas was detected *CLDN1* overexpression [13, 29]. However, its expression pattern and biological function remain largely unknown in the context of human lung adenocarcinoma. Here, we showed that *CLDN3* was frequently upregulated in human ADC, and its upregulation was significantly associated with poor DFS and OS in ADC patients. We also observed that the forced expression of *CLDN3* increased the malignant potential in ADC. Furthermore, We demonstrated that EGF induced the overexpression of *CLDN3* protein, an event mediated by the EGFR downstream MEK/ERK and PI3K/Akt pathways. Moreover, the genetic silencing of *CLDN3* also reduce the tumor burden *in vivo*.

Similar as *CLDN3* overexpression in colonic [30] and ovarian [22] cancers, *CLDN3* expression is increased in most of lung adenocarcinoma. Overexpression of *CLDN3* predicts poor prognosis of ovarian serous adenocarcinoma [31]. Consist with ovarian adenocarcinoma, this study also show that the overexpression of *CLDN3* is significantly correlated with recurrence, metastasis and survival. We also found that *CLDN3* expression in ADC tissue and cells was significantly correlated with E-cadherin, N-cadherin and vimentin expression, and it thus may be involved in the development of ADC. Such changes in EMT marker have been observed in various cancers and experimentally linked to increased cell motility, invasion and metastasis [32]. Furthermore, *CLDN3* knockdown decreased the cell migration and proliferation of A549 and PC9 cells, whereas *CLDN3* overexpression increased H358 cell proliferation, colonization, migration and chemotherapy resistance. The silencing *CLDN3* expression in the ADC cells also decreased tumor growth *in vivo*. Similarly, the upregulation of *CLDN3* in ovarian epithelial cancer enhances matrix metalloproteinase-2 activity and is associated with tumor invasion and angiogenic effects [6]. In contrast, the ectopic expression of *CLDN3* in hepatocellular carcinoma cells could inhibit tumorigenesis and cancerous cells migration and invasion [33]. A recent study revealed that downregulation of *CLDN3* in SqCC patients is an independent prognostic factor for DFS, and correlated with an decreased expression of E-cadherin and β-catenin and an upregulation of vimentin [34]. However, compared to SqCC and normal tissue, an increased expression of *CLDN3* was found in ADC [19–21]. In addition, *CLDN3* expression was also having versatility in breast carcinoma [35–37]. Resolving this discrepancy in different types of tumor cells will require a better understanding of the specific function of different claudin member [29]. These reports suggest that *CLDN3* expression may be involved in organ/tissue specifiity, and the increased expression of *CLDN3* participates in lung adenocarcinoma tumorigenicity [13].

EGFR and its downstream signaling pathways involving MEK/ERK, p38 MAPK, and PI3K/Akt play key roles in the proliferation and differentiation of normal epithelial cells and has been associated with the development of tumor, especially lung cancer [3, 38]. EGFR signaling pathway also modulates fence and barrier production. However, EGF mediated the alternation of claudin protein expression are a great difference and depend on the different cell type and tumor as previous described. For instance, a recent study showed that EGF induced downregulation of *CLDN3* in mucinous cystadenocarcinoma via the MEK/ERK or PI3K/Akt signaling pathway by inducing degradation of the TJ proteins with changes in structures and functions [25]. Conversely, upregulation of *CLDN3* play fundamental role to promote the development of colorectal cancer, which is potentially regulated by the EGF-activated downstream pathway, ERK1/2 and PI3K-Akt signaling pathways [30]. More importantly, the EGF-induced overexpression of *CLDN3* in ADC cells was paralleled by speeding cell proliferation. Furthermore, the cells were pretreated with EGFR and its downstream signaling inhibitors before EGF activation. Interestingly, pretreatment with the inhibitors of EGFR, PI3K/Akt and MEK/ERK decreased the level of *CLDN3* induced by EGF-activation, but not p38 MAPK. The EGF-induced increase in *CLDN3* was eliminated completely upon inhibition of MEK/ERK signaling and partially upon inhibition of PI-3 kinase. Activation of EGF signaling pathway not only induced *CLDN3* expression but also appeared necessary to maintain the baseline *CLDN3* expression. More importantly, downregulation of *CLDN3* abolished this EGF-induced increase in proliferation. These findings demonstrated that EGF mediated the alteration of claudins in ADC cells via the MEK/ERK or PI3K/Akt pathways as modulators of *CLDN3* upregulation-related tumor progression in ADC.

*CLDN3* is one of the natural receptor for the *Clostridium perfringens* enterotoxin, a potent cytolytic toxin. Therefore, CPE-based treatment for malignancies increased *CLDN3* expression, such as ovarian tumor, chemotherapy-resistant ovarian cancer and other aggressive solid tumors may be effective [22, 39, 40]. As one of the most successful treatment in ovarian cancer, *CLDN3* is overexpressed in a majority of ovarian cancer but not detected in normal tissues. Furthermore, other target treatments for *CLDN3*, siRNA therapy or recombinant shRNA plasmid and cisplatin, have been shown to inhibit ovarian cancer progression and reduce the amount of cytotoxic drugs [41–44]. Thus, our study indicates *CLDN3* may be as a molecular target for future application in the ADC treatment.

Overall, we have indicated a consistent and significant increase in *CLDN3* expression in lung adenocarcinoma and a causal association between *CLDN3* overexpression and prognosis of ADC patients. Additionally, we have provided new function of *CLDN3* in the regulation of ADC proliferation and invasion. Importantly, we demonstrated EGF and its downstream signaling pathway MEK/ERK or PI3K/Akt mediated the upregulation of *CLDN3* expression and maintain the basic level of claudins. Furthermore, we showed that *CLDN3* silencing can decrease the malignant potential and prevent the EGF-induced proliferation. Most importantly, our study contributes to a better understanding of the new function of *CLDN3* and the molecular mechanisms that regulate the *CLDN3* expression. Lastly, our study indicates that *CLDN3* became a potential molecular target for future application in the treatment of ADC.

## MATERIALS AND METHODS

### Cell culture and clinical specimens

Lung adenocarcinoma cell lines, including NCI-H1650, NCI-H1299, LTEP-a 2, NCI-H1975, CaLu-3, A549, PG49, NCI-H358, NCI-H1299 and HEY-293T, were obtained from ATCC. The cell lines were maintained in Roswell Park Memorial Institute (RPMI) -1640 containing 10% fetal bovine serum (FBS; Invitrogen, USA).

The lung carcinoma tissues and clinical data obtained from our institute were approved by the Institutional Review Board of China (approval ID 81470137). We also subjected the cell lysates from the clinical specimens to Western blot analysis.

### Western blot

The total protein was extracted, and the concentrations were determined using the Bradford Protein Assay (Bio Rad, Hercules, CA). 50 μg of protein from each sample was separated on 8–12% SDS PAGE and transferred onto polyvinylidene fluoride membrane (Bio Rad). Blots were detected by incubation with antibodies to *CLDN3, CLDN4* (Abcam), EGFR, E-cadherin, Vimentin, N-cadherin, caspase-3, Akt, p-Akt, ERK1/2, p- ERK1/2 and GAPDH (Cell Signaling, Danvers, MA).

### Immunohistochemical analysis

Tumors were removed, weighed, fixed in 5% formalin, and prepared for histological analysis. Immunohistochemical staining was carried out using the ABC staining kit (Santa Cruz Biotechnology; CA, USA) and a secondary biotinylated antibody to rabbit IgG (Invitrogen). *CLDN3*, *CLDN4*, EGFR, E-cadherin, Vimentin, and N-cadherin immunoreactivity were assessed based on a combined score of the extent and intensity of staining. Scores 0–3 were assigned according to the percentage of positive tumor cells (0=0%; 1=<33%; 3=33–66%; 3=>66%) and the intensity of tumor staining (0=0; 1=1+; 3=2+; 3=3+). The two scores were multiplied to give an overall score of 0–9, for which 0 was considered negative, 1–2 was considered weak, 3–6 was considered moderate, and 9 was considered strong staining. Negative and weak expression was considered to be low, whereas moderate and strong expression was considered to be high. Any discordant scores were reviewed together by both scorers to obtain a consensus score.

### Lentivirus-based short hairpin RNA transduction

Short hairpin RNA (shRNA) lentiviral transduction particles for the *CLDN3* knockdown experiments were obtained from Sigma (St Louis, MO). Two of three *CLDN3*-specific shRNA constructs (shRNA sequence targeting *CLDN3*: CCGGCGACCGCAAGGACTACGTCTACTCGAGTAGACGTAGTCCTTGCGGTCGTTTTTG; CCGGGACTACGTCTAAGGGACAGACCTCGAGGTCTGTCCCTTAGACGTAGTCTTTTTTG) and one “nontarget” construct were transduced separately into 293T cells. The “nontarget” construct contained a shRNA sequence that did not target any known human gene and served as a scrambled negative control. Briefly, 293T cells were transduced with the *CLDN3* specific shRNA lentiviral particles for 24 hours in the presence of hexadimethrine bromide to improve the transduction efficiency. Afterward, the medium containing viral particles was removed and replaced with fresh medium containing 10 μg/ml puromycin. Before lentiviral transduction, a puromycin titration was performed that identified 10 μg/ml as the minimum puromycin concentration to cause the complete death of the 293T cells after a 5-day incubation. *CLDN3* knockdown was confirmed using a Western blot analysis based comparison to the nontarget shRNA cells.

### NCI-H1975^Cld3^ and NCI-H358^Cld3^ cell construction

NCI-H1975^Cld3^, NCI-H358^Cld3^ or empty-vector (NCI-H1975pCDH, NCI-H358pCDH) cells were generated by transducing NCI-H1975 and NCI-H358 wild-type cells with pCDH-Cld3 or empty vectors and selecting successfully transduced cells with 7.5 mg/mL puromycin for at least 5 days. The clones were isolated, and the overexpression of *CLDN3* and 4 was confirmed by immunoblotting.

### Fluorescence confocal microscopy

NCI-H1975 and NCI-H358 cells were transfected with a control or pCDH-Cld3 for 72 h. Cells were fixed in 4% formaldehyde and subjected to indirect immunofluorescence microscopy with anti-*CLDN3*. Confocal immunofluorescence microscopy (Olympus; Tokyo, Japan) was performed using an Olympus confocal microscope according to the manufacturer's protocol. The magnification used was 40×.

### Cell proliferation assay

After cells were seeded in 24-well plate, EdU was added to the culture medium at a concentration of 50 μM/ml for 8 h to chase the DNA template according to the instructions in the Cell Light EdU DNA cell kit (Apollo 488/567, RiboBio, China). Briefly, after being fixed in 4% paraformaldehyde and treated with 0.5% Triton X for 15 min, cells were incubated in darkness with Apollo, and their nuclei were stained by Hoechst 33342. EdU-labeled cells were counted manually in five fields of view randomly selected from each well, and the percentages were calculated.

### Anchorage-dependent colony formation

For the anchorage-dependent colony formation assays, A549(Cld3KD), PC9 (Cld3KD), NCI-H358^Cld3^ and the relevant controls were seeded in a six-well plate at a low density (5×10^2^) for 7 days. After 7 days, the cells were fixed with ethanol for 6 min and stained with a crystal violet solution (0.05% crystal violet and 20% methanol). The cells were then washed twice with water and solubilized with methanol. All experiments were performed in triplicate.

### Wound scratch assay

Each group of A549 (Cld3KD), PC9 (Cld3KD), NCI-H358^Cld3^ and the relevant controls were seeded into a six-well plate. On the following day, when the cells were approximately 90% or more confluent, each well was scraped with a 20 μl pipette tip to create 3 linear regions devoid of cells. Then, the cells in each well were cultured with RPMI-1640 medium (Gibco, USA) containing 2% fetal bovine serum (Gibco, USA) in a humidified incubator. Photographs of the wounded area were taken immediately after making the scratch (0 hour time point) and after 24 hours to monitor the invasion of cells into the wounded area.

### Transwell tumor cell migration assay

A 24-well Boyden chamber with an 8 μm pore size polycarbonate membrane (Corning, NY) was used to evaluate cell motility. 10^5^ cells were seeded in the upper chamber with 200 μl serum free medium. 600 μl medium with 10% serum was added into the lower chamber as a chemoattractant. 16 h after incubation, the membranes was fixed with methanol and stained with a three-step staining set (Thermo, UK). Five visual fields were randomly selected from each membrane. All experiments were performed in triplicate.

### Treatment with EGF and pharmacological inhibitors

Cell cultures were treated with 100 ng/mL EGF, a concentration consistent with that used in other studies [26]. The effects of EGF treatment on the *CLDN3* and *CLDN*4 expression in A549 and H358 cells were assessed in cells growing in RPMI-1640 medium supplemented with 10% FBS after 48 h. For pharmacological inhibition, cells were serum starved overnight, and selective inhibitors were added to the cell cultures 1 h before EGF treatment to inhibit the intrinsic kinase activity. The cells were then incubated with fresh culture medium supplemented with 10% FBS containing EGF and selective inhibitors, which were maintained throughout the experiments. The inhibitors were diluted in DMSO and stored at -20°C. Each concentrated solution was diluted immediately before each experiment to yield final concentrations of 0.5 μM (PD153035; EGFR inhibitor), 10 μM (U0126; ERK1/2 kinase inhibitor), 20 μM (LY294002; PI -3 kinase inhibitor) or 5 μM (SB239063; p38 kinase inhibitor) [26].

### *In vivo* tumorigenicity

1×10^7^ stable A549 (Cld3KD) cells and control cells were injected subcutaneously into the left groin of 6-week-old male BALB/c nude mice (n=7 per group). The tumor diameter was measured every 3 days from the 28th day after inoculation for 25 days. The tumor volume was calculated by the formula V=0.5×L×W^2^. All animal experiments were approved by the Animal Experimentation Ethics Committee of the Tianjin medical university cancer hospital and institute.

### Statistical analysis

Associations between the *CLDN3* expression and clinical and biological characteristics were analyzed by x^2^ or Fisher's exact test. Survival curves were drawn using the Kaplan–Meier method. The Cox proportional hazards regression (forward likelihood ratio model) was used for multivariate survival analyses. All two-group comparisons utilized Student's t-test with the assumption of unequal variance. Data are presented as the mean ± SEM determined from a minimum of 3 independent experiments. All data were analyzed using the Statistical Package for the Social Sciences Version 18.0 Software (SPSS Inc., Chicago, IL). The two-sided significance level was set at *p* <0.05.
